# Snakebites by *Bothrops taeniatus*: characteristics of snakebites involving a rare venomous snake in Brazil

**DOI:** 10.31744/einstein_journal/2025RC1430

**Published:** 2025-10-20

**Authors:** Paula Cristina Yukari Suzaki Fujii, Bruna Seffrin Soares, Eduarda Scoto Dias, Henrique Lira Borges, Daniel Emilio Dalledone Siqueira, Ramon Cavalcanti Ceschim

**Affiliations:** 1 Faculdade Pequeno Príncipe Curitiba PR Brazil Faculdade Pequeno Príncipe, Curitiba, PR, Brazil.; 2 Faculdade Evangélica Mackenzie do Paraná Curitiba PR Brazil Faculdade Evangélica Mackenzie do Paraná, Curitiba, PR, Brazil.; 3 Secretaria de Estado da Saúde do Paraná Centro de Informação e Assistência Toxicológica do Paraná Curitiba PR Brazil Centro de Informação e Assistência Toxicológica do Paraná, Secretaria de Estado da Saúde do Paraná, Curitiba, PR, Brazil.

**Keywords:** Bothrops taeniatus, Snake bites, Snake venoms, Antivenins, Epidemiology

## Abstract

Snakebites from the genus *Bothrops* account for up to 90% of venomous snakebites in Brazil. Among these species, *Bothrops taeniatus* is considered rare in Brazil, with only one reported case in the literature. Its venom has a strong hemorrhagic effect but lacks procoagulant activity, which can lead to significant variations in the neutralizing efficacy of anti-Bothrops serum. We describe the first case of severe envenomation by *B. taeniatus* with rapid recovery following the administration of anti-Bothrops serum in Brazil, and discuss the characteristics of *B. taeniatus* envenomation based on a literature review.

## INTRODUCTION

Snakebites caused by the genus *Bothrops* are a significant public health issue in Brazil, accounting for approximately 90% of all venomous snakebites in certain regions.^(1)^ Among the more than 30 species within the genus, *Bothrops taeniatus*, also referred to as *Bothriopsis taeniata*, *Bothrops lichenosa*, or *Bothrops castelnaudi*, and commonly known as the Speckled Forest-Pitviper, or *Jararaca-estrela* and *Jararaca-cinza* in Brazil, is considered a rare snake, particularly in Brazil, with only one reported envenomation case described in the literature by Torrez et al.^(2)^

Native to the Amazon region, its distribution extends to Ecuador, Colombia, Venezuela, Peru, Bolivia, Guyana, Suriname, and French Guiana.^(3)^ In Brazil, envenomation has been reported in the states of Acre, Amazonas, Maranhão, Mato Grosso, Pará, Roraima, Rondônia and Tocantins.^(4)^ This is the first report of severe envenomation by this species with rapid recovery following antivenom therapy in Brazil. A literature search and review were conducted in the LILACS, MEDLINE, PubMed, and SciELO databases.

## CASE REPORT

A 14-year-old male resident of Carlinda, Mato Grosso (MT), Central-West Brazil, reported being bitten by a snake on his left ankle while in the chicken coop of his rural residence at approximately 20:00 h.

**Figure 1:**
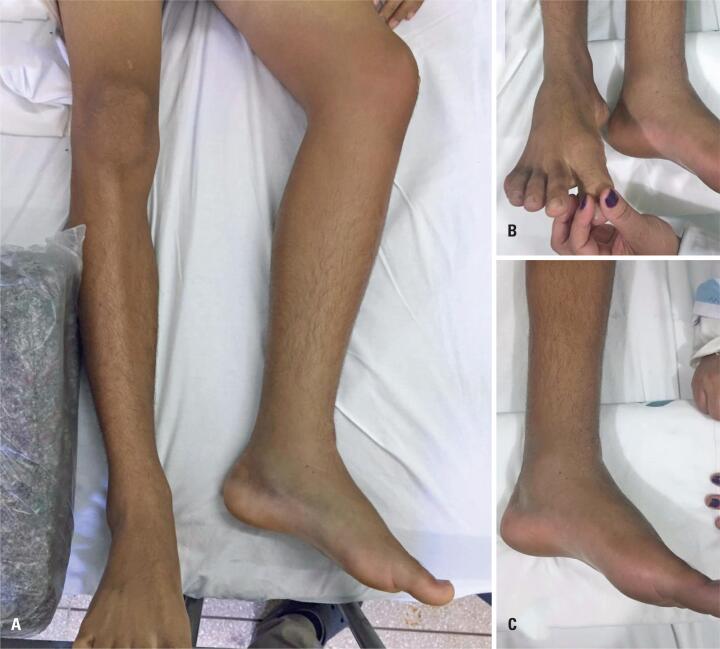
Bite site at the time of hospital admission, approximately 2 h after the bite; (A): Lower limbs comparing swelling; (B): Left ankle (location of the bite) comparing swelling; (C): Left foot swollen from the bite

Approximately 2 h after the bite, the patient sought medical care in the nearby city of Alta Floresta, Brazil. Upon admission, the patient was hemodynamically stable but reported moderate pain and rapidly progressive swelling in the ankle and distal third of the left leg (Figure 1).

**Figure 2:**
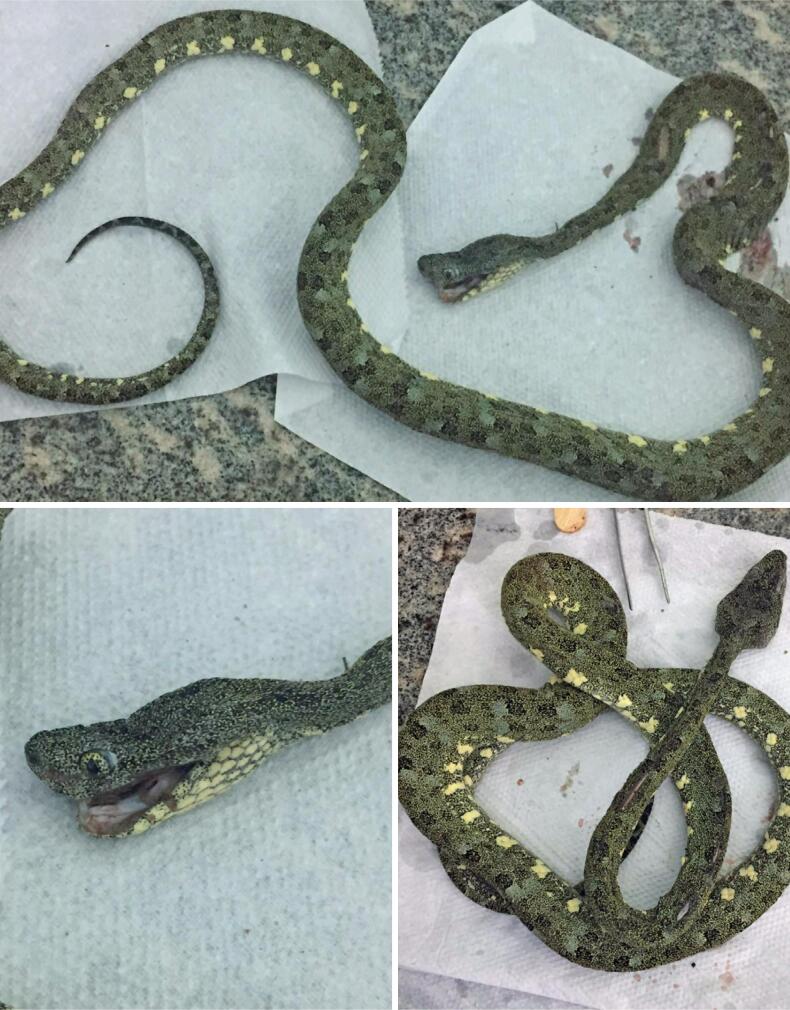
The snake that bit the patient identified as *Bothrops taeniatus*

After contacting the Toxicological Information and Assistance Center of the State of Paraná (CIATox-PR), a possible bothropic envenomation was discussed, which was later confirmed to be caused by *B. taeniatus* following identification by a CIATox biologist (Figure 2).

The severity of snakebite envenomation was initially classified as mild, and general guidelines were provided for the initial management of snakebites and antivenom therapy following the protocol of the Brazilian Ministry of Health, which recommends the administration of three ampoules of anti-Bothrops serum (ABS).

The next morning, contact with the hospital revealed that 12 ampoules of ABS had been administered during the night, not clearly indicating the time of administration but between 1 and 6 h of hospital admission. The patient remained stable after antivenom therapy; however, he developed extensive edema extending to the thigh, experienced pain during limb movements, and reported three episodes of vomiting. Laboratory tests conducted at admission and 12 h after completion of antivenom therapy showed no significant abnormalities. Given the progression of swelling and pain, the patient's condition was reclassified as severe. Approximately 36 h after admission, the patient was discharged due to significant clinical improvement, with resolution of vomiting and reduction of swelling and pain, reporting only mild local swelling at discharge but no laboratory changes. No complications were reported during hospitalization. At follow-up, the patient showed good clinical progress, with remission of signs and symptoms.

This study was approved by the Research Ethics Committee of *Hospital do Trabalhador / Secretaria de Estado da Saúde do Paraná* (CAAE: 81316224.7.0000.5225; #7.002.901).

**Table 1:** Progression of the patient's laboratory test during hospitalization

Laboratory test	At hospital admission	12 h after administration of ABS
Hemoglobin (13.2–18.0g/dL)*	14.3g/dL	13.3g/dL
Hematocrit (39.0–51.0%)	42.1%	40.3%
Segmented (45.5–74 %)	78%	89%
Leukocytes (3800–11000mm^3^)	11.100mm^3^	7.700mm^3^
Platelet (150.000–440.000mm^3^)	160.000mm^3^	155.000mm^3^
Urea (15–39mg/dL)	28mg/dL	27mg/dL
Creatinine (0.8–1.3mg/dL)	0.82mg/dL	0.96mg/dL
INR (less than 1.3)	1.36	1.36
Prothrombin time (10–16.6 s)	16.4 s	14.2 s
Coagulation time (up to 10 min)	-	2.32 min

* The normal range of each parameter is specified in parentheses. INR: international normalized ratio.

ABS: anti-Bothrops serum.

## DISCUSSION

*Bothrops taeniatus* is considered an arboreal and nocturnal snake that, unlike other species of the same genus, does not exhibit a dorsal body pattern resembling an inverted V. Its general dorsal coloration can vary from light brownish-gray to greenish-yellow^(4)^ making it difficult to identify. Owing to the cryptic resemblance of the body to leaves and lichens, identification at the time of envenomation is uncommon, which explains the low number of confirmed cases.^(2)^

Similar to other bothropic envenomations, the severity is determined by the patient's clinical condition, including local symptoms, presence of bleeding, coagulation disorders, swelling, and complications, which guide case management.^(5)^

In mild cases, pain and swelling were observed in up to one segment of the body, whereas in moderate cases, they were observed in up to two segments. Hemorrhage or coagulopathy may be present or absent, and the criteria for classification are not mandatory for evaluating the need for treatment with additional ABS vials. In severe cases, pain and swelling are found in three segments, or there may be systemic complications, such as severe hemorrhage, hypotension/shock, and acute renal injury.^(5,6)^

Anti-Bothrops serum is a specific treatment for envenomation caused by snakes of the *Bothrops* genus, with the number of ABS ampoules administered depending on the severity of the envenomation (mild, moderate, or severe).^(5,6)^ The effectiveness of the antivenom therapy was monitored by performing coagulation tests 12 and 24 h after administration to assess the need for additional ampoules.^(6)^ In this particular case, the rapid progression of swelling indicated worsening of the condition, revealing the need for re-stadiation and, consequently, an increase in the number of ABS vials for treatment.

In Brazil, ABS is produced by hyperimmunizing horses with venom pools of *Bothrops alternatus* (12.5%), *Bothrops jararaca* (50%), *Bothrops jararacussu* (12.5%), *Bothrops moojeni* (12.5%), and *Bothrops neudi* (12.5%). This formulation considers the immunogenic potential in horses, the frequency of snakebites, and the geographic distribution of species with the aim of neutralizing all *Bothrops* envenomations.^(7–9)^

Traditionally, the neutralizing efficacy of anti-Bothrops antivenom has been assessed based on its ability to inhibit the lethal activity of *B. jararaca* venom, which is considered the reference venom.^(9)^ However, despite the broad epitopic cross-reactivity among Viperidae species, there are peculiarities that may significantly affect serum neutralization efficacy, especially in envenomations caused by less common species such as *B. taeniatus.*^(7)^

Although *Bothrops* venoms are characterized by their effects on three main pathways – proteolytic, hemorrhagic, and coagulant – coagulant, necrotizing, and myotoxic activities are not equally distributed among species.^(10)^ Variations in venom composition and the effectiveness of ABS depend on several factors including the phylogenetic characteristics of the species, seasonality, geographic distribution, and ontogenetic stage of the snake. Even species within the same genus or individuals of the same species can differ in the quantity and quality of toxins present.^(7)^ Therefore, it is crucial to understand the differences in venom composition and ABS efficacy for specific species such as *B. taeniatus*.

Muniz et. al.^(9)^ demonstrated that seroneutralization tests conducted in vitro and on mice against the venoms of *B. jararaca*, *B. atrox*, *B. brazili*, *B. bilineatus smaragdinus*, and *B. taeniatus*, produced significant variation in the neutralizing efficacy of ABS. Among the species tested, *B. taeniatus* venom exhibited the highest lethality and the dose required to neutralize *B. taeniatus* venom was greater than that required for *B. jararaca* venom, the reference venom used for assessing antibothropic serum potency in Brazil. Despite the good response of the case reported to treatment, there was a need for re-staging due to progression of the condition, raising discussion regarding the therapeutic approach and number of vials of ABS required, depending on the known lower response to ABS with respect to the species of *Bothrops* involved.

In another mouse study, Furtado et al.^(7)^ compared the properties of the venom of *B. atrox* and *B. taeniatus*. Similarly, *B. taeniatus* venom was found to be the most toxic, showing a faster onset of activity, peaking at 16 h post-inoculation compared to 24 h for the other species, and causing significantly higher hemorrhagic activity. The study highlighted that the minimum hemorrhagic dose, the dose that induces a 10mm diameter lesion, was nearly 10 times lower for *B. taeniatus* than for the other species.

Unlike other species of the same genus, *B. taeniatus* venom did not inhibit blood coagulation in mice when injected intravenously, indicating a lack of procoagulant activity, but a strong hemorrhagic effect. This finding supports those of Kuch et al.,^(11)^ Kamiguti et al.^(12)^ and Freitas-de-Sousa et al.,^(3)^ which also highlight the absence of coagulant activity in *B. taeniatus* venom. It has been suggested that the venom exerts its anticoagulant effect through the inactivation and inhibition of factor X activity and the direct inhibition of thrombin's coagulant function.^(12)^ In transcriptomic and proteomic analyses of *B. taeniatus* venom, the very low catalytic activity of snake venom serine proteinases found in the venom explains this observation.^(3)^

Freitas-de-Sousa et al.^(3)^ also noted that venom contains high proportions of C-type lectin-like proteins (CTLs), which may cause hemostatic disturbances by activating platelets, potentially leading to platelet depletion, which is further exacerbated by anticoagulation, as CTLs can be potent thrombin inhibitors. Additionally, the dominant acidic isoform of phospholipase A2 in venom predicts anticoagulant activity, whereas a low proportion of basic isoforms suggests a very low level of myotoxicity.

Notably, despite these venom characteristics, no laboratory findings related to coagulopathy were observed in either the first Brazilian case described or the current case. Despite the strong hemorrhagic action described in the literature, none of the patients presented with significant clinical signs of bleeding. Although not mandatory, the criteria for staging, bleeding, and laboratory changes in coagulation are important adjuvants in the diagnosis of *Bothrops* accidents, especially when the agent is not identified, helping in the correct therapeutic approach for cases. However, it is important to consider the possibility of bothropic accidents without coagulation abnormalities in laboratory tests or marked clinical signs of bleeding, as evidenced in these two cases. Such scenarios highlight the potential underestimation of severity in cases in which traditional markers of envenomation are absent.

In conclusion, because of the difficulty in identifying *B. taeniatus*, largely because of its camouflage, which means that it is rarely used for identification, clinical presentation and laboratory tests have become critical diagnostic tools. The variations in the composition of *B. taeniatus* venom and their impact on the patient's clinical condition underscore the importance of recognizing these specificities, which may obscure the typical clinical and laboratory findings observed in envenomations caused by other *Bothrops* species, thereby complicating the assessment of severity. This emphasizes the need for increased clinical vigilance in managing envenomation caused by less-common species.

The importance of toxicological information centers is also emphasized given the challenges in identifying the venomous species involved, which can lead to delayed treatment and unfavorable outcomes. Assistance with species identification and guidance on therapeutic measures enables effective management and reduces the likelihood of complications and poor outcomes.
